# Ultrasonographic characteristics of renal lymphoma in cats receiving chemotherapy

**DOI:** 10.1177/1098612X251393542

**Published:** 2025-10-24

**Authors:** Alessia Cordella, Chantelle Franklin, Helen Dirrig, Stefano De Arcangeli, Jennifer Lenz

**Affiliations:** 1 Department of Clinical Sciences and Advanced Medicine, University of Pennsylvania School of Veterinary Medicine, Philadelphia, PA, USA; 2 Department of Clinical Science and Services, The Royal Veterinary College, London, UK; 3 VetCT Consultants in Telemedicine, Adelaide, South Australia, Australia

**Keywords:** Kidney, lymphosarcoma, oncology, azotemia, subcapsular rim

## Abstract

**Objectives:**

The aim of this study was to describe renal ultrasonographic (US) findings in cats with confirmed renal lymphoma receiving chemotherapy and correlate them with clinical and clinicopathological findings.

**Methods:**

For this multicenter retrospective study, cats were included if they had cyto-/histological confirmation of renal lymphoma, received multiagent chemotherapy, and US images of the kidneys before and after treatment were available. All images at T0 (diagnosis) and T1 (after chemotherapy) were reviewed. Oncology records were reviewed, and serum creatinine and blood urea nitrogen (BUN) concentrations were recorded, when available.

**Results:**

A total of 24 cats (20 males, 4 females; median age 8 years) who underwent vincristine, cyclophosphamide and prednisolone (COP)-based chemotherapy were included. At T1 (median 33 days, range 20–60), 21 cats were considered to have experienced clinical benefit (20 with improved renal appearance on ultrasound, one with static appearance), two cats had stable clinical findings (one stable, one progressive ultrasound) and one cat clinically declined (progressive ultrasound). On ultrasonography, nephromegaly resolved in 12/20 cats, hypoechoic subcapsular rim disappeared in 6/17 and reduced in 7/17, and nodules and/or masses disappeared in 8/14 and reduced in 5/14 cats. Six cats had normal creatinine and BUN concentrations at both T0 and T1 (five improved and one with stable ultrasound findings); in 11 cats, the azotemia detected at T0 resolved at T1; four cats were persistently azotemic (all with reduced creatinine and BUN concentrations) and one cat became azotemic on T1 (progressive ultrasonography).

**Conclusions and relevance:**

After chemotherapy, kidneys affected by lymphoma commonly returned to their regular size; subcapsular rim, nodules and masses markedly reduced or completely resolved. US findings were often in agreement with clinical and clinicopathological findings.

## Introduction

Lymphoma is the most common renal neoplasm in cats, with a reported incidence of renal involvement in cases of feline lymphoma in previous studies in the range of 6–30%, and can occur as a single entity or as a part of multicentric disease.^1–6^

Abdominal ultrasound is considered the modality of choice to image the urinary tract in cats. It is a useful and non-invasive technique to assess the renal size, shape, contour and internal architecture as well as the appearance of the retroperitoneal space.^7,8^

Nephromegaly is the most common ultrasonographic (US) characteristic described in feline renal lymphoma, alongside increased cortical echogenicity, hypoechoic subcapsular thickening, single or multiple nodules and pyelectasia.^6,8–11^ US guidance is also commonly used to obtain samples for cytology evaluation; renal lymphoma often exfoliates readily, typically resulting in high cellularity.^12^ Cytology usually reveals sheets of monomorphic lymphocytes with high nuclear:cytoplasmic ratio and a scant amount of basophilic cytoplasm.^12^

The preferred treatment for renal lymphoma consists of multidrug chemotherapy protocols, with a variety of different protocols and durations and response rates reported.^13–17^ Commonly recommended protocols to treat lymphoma in cats often include combinations of vincristine, cyclophosphamide and prednisolone (COP) at different intervals. Doxorubicin is often omitted from protocols because of the risk of nephrotoxicity and lower response rates reported in cats.^18–20^

Despite ultrasonography being commonly used to assess response to treatment in feline patients with renal lymphoma, studies describing the appearance of kidneys after chemotherapy are currently lacking. Therefore, the aims of this study were to describe renal US features at diagnosis and after initiating chemotherapy in cats with renal lymphoma and to compare renal US appearance with clinical and clinicopathological findings.

## Materials and methods

For this retrospective descriptive study, the electronic medical records of cats examined at the Ryan Veterinary Hospital (School of Veterinary Medicine, University of Pennsylvania) and Queen Mother Hospital for Animals (Royal Veterinary College, University of London) between 2012 and 2024 were reviewed. Cats were included if they had a cytological or histological confirmation of renal lymphoma and US images of the kidneys (at least two images of each kidney at each time point) before and after initiation of chemotherapy available for review, and if details on the type of chemotherapy protocol used were available.

Type of lymphoma, when specified by the cytology report, was recorded. All cytology reports were performed by board-certified clinical pathologists (American College of Veterinary Pathologists-ClinPath or European Society of Veterinary Clinical Pathology Diplomates). Cats with uncertain diagnosis from the cytology report (including malignant neoplasia, atypical cells and round cell tumors) were excluded. Cats were also excluded if they had incomplete images of the kidneys at one or more time points, if the diagnosis of renal lymphoma was not definitively confirmed (cases of lymphoma diagnosed by cytology on other organs but not specifically on kidneys) or if they underwent chemotherapy for lymphoma before the first US examination. Cats with no recorded US follow-up were also excluded.

Signalment (breed, age at time of diagnosis and sex) and presumed (on imaging) or confirmed (on cytology and/or histology) involvement of other organs were also recorded for each case.

When clinical chemistry was available at the same date as the US examination, the concentration of serum creatinine and blood urea nitrogen (BUN) were recorded. Cats were classified as azotemic or non-azotemic based on the reference intervals (RIs) provided by the laboratory where the biochemical analysis was performed. Medical records for each cat were reviewed by a board-certified oncologist, American College of Veterinary Internal Medicine (Oncology) Diplomate (JL), to determine the chemotherapy protocol used and to assign a clinical response to treatment at each imaging time point during chemotherapy without knowledge of the corresponding imaging results. Cats were considered to have experienced positive clinical benefit if owners reported improved clinical signs compared with baseline and patients had increased weight or reduced kidney size on physical examination. Cats with clinical decline experienced worsening clinical signs, weight loss or progressive nephromegaly on examination. If patients did not easily fall into either category or had conflicting clinical findings, they were considered to have static clinical disease.

### Ultrasound image review

All US images of the kidneys were reviewed by a board-certified radiologist, European College of Veterinary Diagnostic Imaging Diplomate (AC), aware of the diagnosis of lymphoma but unaware of the clinical and clinicopathological results. Recorded US findings included, for each kidney, presence or absence of the following: nephromegaly, defined as maximum renal length >44 mm,^21^ hypoechoic subcapsular rim,^9^ pelvic distension, perinephric effusion and steatitis (hyperechogenicity of the perinephric tissues). The pelvis was considered distended when visible and measurable, and dimension was measured (in mm) in a transverse plane.^22^ The presence of a single mass or multiple masses and/or nodules was also recorded, with evaluation of dimensions (in mm) of the largest one and echogenicity (hypo-, iso- or hyperechoic compared with cortical parenchyma).

For each cat, US examinations were divided into time points T0 (time of diagnosis) and T1 (first US follow-up after chemotherapy initiation). After independently reviewing all T0 and T1 images and recording the US findings, T0 and T1 were compared for each cat. Based on the US findings (modified from Response Evaluation Criteria in Solid Tumors guidelines^23^), the cats were classified as follows: (1) improved, if appreciable changes were noticed in at least one of the following: decreased renal size, decreased or resolved number and/or size of the masses, or decreased or resolved hypoechoic subcapsular rim; (2) stable, if no appreciable changes were observed; and (3) progressive, if renal size was increased, masses were more numerous or larger, or the hypoechoic subcapsular rim was thicker or newly observed. Perinephric effusion and/or steatitis and renal pelvis distension were also evaluated (resolved, improved, static or worsened); however, these findings alone were not sufficient to classify the disease as improved, stable or progressive – they were considered only in conjunction with primary renal findings. For cases with than one available follow-up, all US examinations after T1 were reviewed chronologically and compared with T1 images using the same criteria.

### Statistical analysis

Statistical analysis was performed using STATA version 18 (StataCorp). For continuous variables measured at T0 and T1, the paired Wilcoxon signed-rank test was used (selected for the relatively small sample size). For paired binary outcomes, McNemar’s χ^2^ test was used to assess the changes in frequency between T0 and T1.

Statistical significance was set at *P* <0.05. Exact *P* values were reported for McNemar’s test to account for the small sample size.

## Results

A total of 24 cats met the inclusion criteria. Of these, 20 (83%) were males (18 castrated) and four (17%) were females (all spayed); the median age at diagnosis was 8 years (range 1.5–14). Most of the cats were domestic shorthairs (n = 18, 75%); other included breeds were Siamese (n = 3, 12.5%), domestic longhair, Bengal and Maine Coon (n = 1 each).

All cats were diagnosed with renal lymphoma as per the inclusion criteria; in 15/24 (62.5%) cases, the lymphoma was classified as large cell, one with Mott cell differentiation. The remaining nine cases had intermediate (n = 2) or intermediate-to-large (n = 7) lymphoma. In one case, lymphoma was highly suspected on renal cytology, and large cell lymphoma was confirmed at necropsy 2 months later.

In 15 (62.5%) cases, one or more other abdominal organs were suspected (on imaging) or confirmed (by cytology) to be involved in the neoplastic process: gastrointestinal tract in 12 cases, lymph nodes in three cases, and liver and spleen in two cases. One cat was diagnosed with both nasal and renal lymphoma at the same time.

A COP-based chemotherapy protocol used for all cats; in most patients (20/24), cytarabine was added to the protocol.

### US findings at the time of diagnosis (T0)

**Figure 1:**
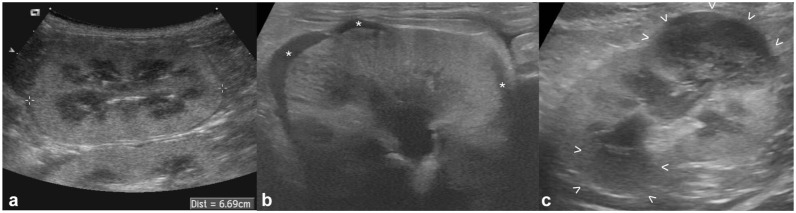
Ultrasonographic appearance of confirmed renal lymphoma at diagnosis (T0) in three cats: (a) marked nephromegaly: the caliper is measuring the renal length at 66.9 mm (range of normality is up to 44 mm); (b) hypoechoic subcapsular rim: crescent-shaped hypoechoic subcapsular thickening is seen in multiple portions of the kidney (*); and (c) renal nodules/masses: two well-defined, hypoechoic masses are visible at the cranial and caudal poles (between arrowheads)

The renal changes detected on US examination were bilateral in 21/24 (87.5%) cats and unilateral in three (12.5%) cats. Most (83%) of the included cats presented with nephromegaly at the time of diagnosis (Figure 1a). The median renal length at T0 was 57 mm (range 42–78). Other common US findings were hypoechoic subcapsular rim (71%) (Figure 1b) and multiple parenchymal nodules and/or masses (42%) (Figure 1c). A single nodule/mass was detected in four (17%) cases.

The median largest diameter of the detected nodules/masses at T0 was 17 mm (range 7–47) and the median pelvic diameter, when measurable, was 2 mm (range 1–9). All detected masses and nodules were hypoechoic (mildly to markedly) compared with the cortical parenchyma.

### US findings after chemotherapy was initiated (T1)

Of the 24 included cats, 21 had an US examination of the urinary tract available for review 4–6 weeks after the chemotherapy was initiated, while in the remaining three cats, the first follow-up was at 2 months (median 33 days, range 20–60).

At the first follow-up after initiation of chemotherapy, the most frequent US finding was the hypoechoic subcapsular rim (n = 11, 46%). Only eight (33%) cats presented with nephromegaly and six with multiple nodules. The median renal length at T1 was 41 mm (range 37–56). The median largest diameter of the detected nodules and masses at T1 was 7.5 mm (range 1.5–17) and the median pelvic diameter was 1 mm (range 1–4).

The frequency of US findings at T1, compared with the frequency of the findings at T0, is summarized in Table 1.

**Table 1:** Renal ultrasonographic findings of the 24 included cats at the time of diagnosis (T0) and at first follow-up after initiating chemotherapy (T1)

Ultrasonographic findings	T0 (n = 24)	T1 (n = 24)	*P*
Nephromegaly	20 (83)	8 (33)	**<0.001**
Presence of a single mass	4 (17)	1 (4)	0.25
Presence of multiple nodules/masses	10 (42)	6* (25)	0.21
Hypoechoic subcapsular rim	17 (71)	11 (46)	**0.03**
Pelvic distension	14 (58)	8 (33)	0.07
Perinephric effusion/steatitis	12 (50)	8 (33)	0.21

Data are n (%). Statistically significant findings (*P* <0.05) are shown in bold

* In one case, nodules were not present at T0 but were visible at T1

A statistically significant difference was observed in the frequency of nephromegaly (*P* <0.001) and hypoechoic subcapsular rim (*P* = 0.03) at T0 and T1.

The renal length and the maximum dimension of the nodules/masses were significantly reduced between T0 and T1 (*P* <0.001 and *P* = 0.03, respectively), while the pelvic diameter did not show statistical significance (*P* = 0.4).

### US and clinical improvement

Based on comparison between US images at T0 and T1, 20/24 (83%) cats were considered improved at T1, two were stable and two were progressive.

**Figure 2:**
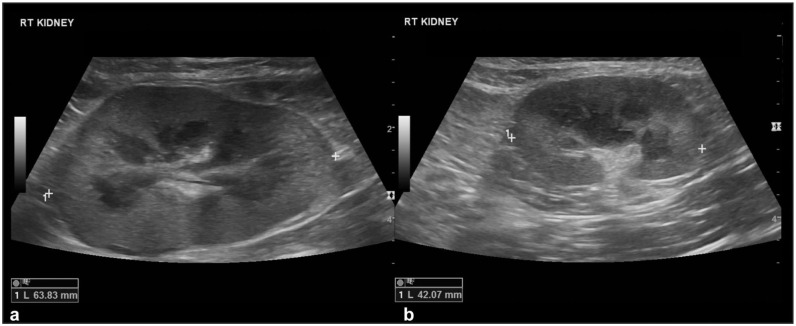
Resolved nephromegaly: (a) right kidney at T0, characterized by marked nephromegaly (caliper measuring 63.8 mm) – a thin irregular hypoechoic subcapsular rim is also present, and the retroperitoneal fat around the kidney is hyperechoic; and (b) the same kidney at T1 (30 days after the first ultrasound) showing normal size (caliper 42 mm) – the subcapsular rim and retroperitoneal steatitis are also no longer present

**Figure 3:**
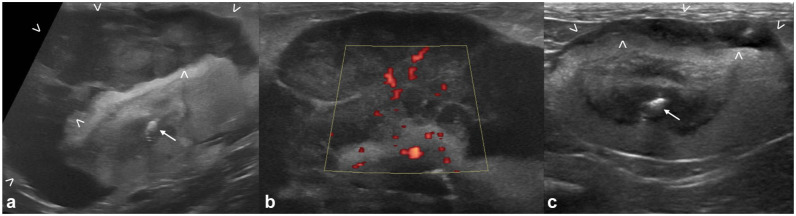
Improved subcapsular rim: (a) appearance of the kidney at T0 with a large mass-like hypoechoic subcapsular thickening (between arrowheads) – a hyperechoic mildly shadowing structure (mineralization/nephrolith) is visible in the region of the renal pelvis (arrow); (b) close-up of the power Doppler interrogation on the mass-like subcapsular hypoechoic thickening at T0 – the hypoechoic tissue is markedly vascularized; and (c) appearance of the same kidney at T1 (45 days after T0) – the subcapsular thickening, still present and hypoechoic, is markedly reduced (between arrowheads). Static mineralization/nephrolith (arrow)

**Figure 4:**
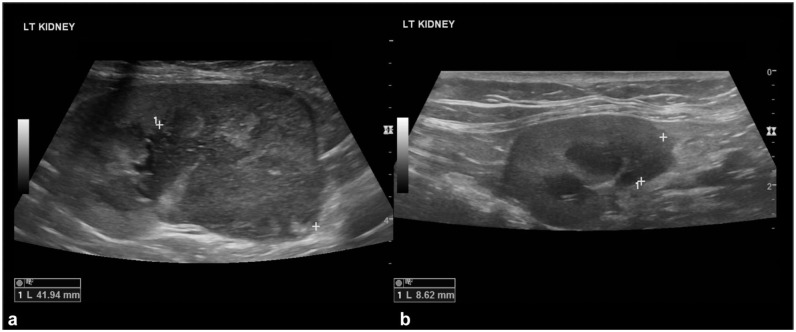
Reduced mass: (a) a large, heterogeneously hypoechoic mass is visible at the caudal pole of the kidney at T0 (caliper measuring 41.9 mm); and (b) the same kidney at T1 (30 days after T0) showing marked reduction of the previously noted mass at the caudal pole (caliper measuring 8.6 mm)

**Figure 5:**
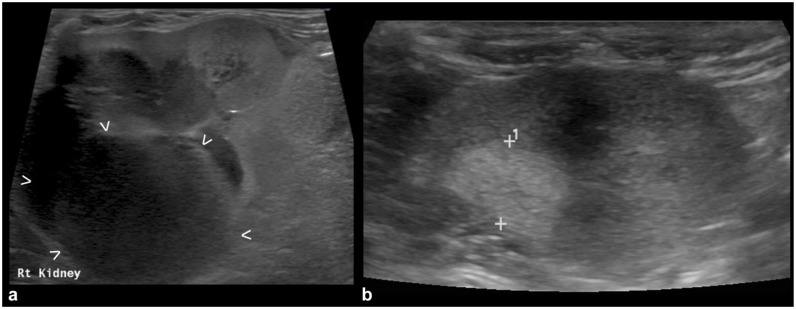
Hypoechoic masses at T0, hyperechoic at T1: (a) kidney at T0, showing a large, heterogeneously hypoechoic mass deforming the renal profile (between arrowheads); and (b) the same kidney at T1 (41 days after T0) – where the hypoechoic mass was noted at T0, a hyperechoic nodule is present at T1 (between calipers)

The nephromegaly resolved in 12/20 (60%) cats (Figure 2), the hypoechoic subcapsular rim disappeared in 6/17 (35%) cases and reduced its size in an additional seven cases (Figure 3), nodules/masses disappeared in 8/14 (57%) cases and reduced in size in an additional five cases (Figure 4). In all but two cats, the nodules and/or masses were hypoechoic at both T0 and T1. In these remaining cats, the detected nodules and/or masses were hypoechoic at T0; at T1, smaller hyperechoic nodules were detected in the regions where the large hypoechoic nodules were at T0 (Figure 5).

**Figure 6:**
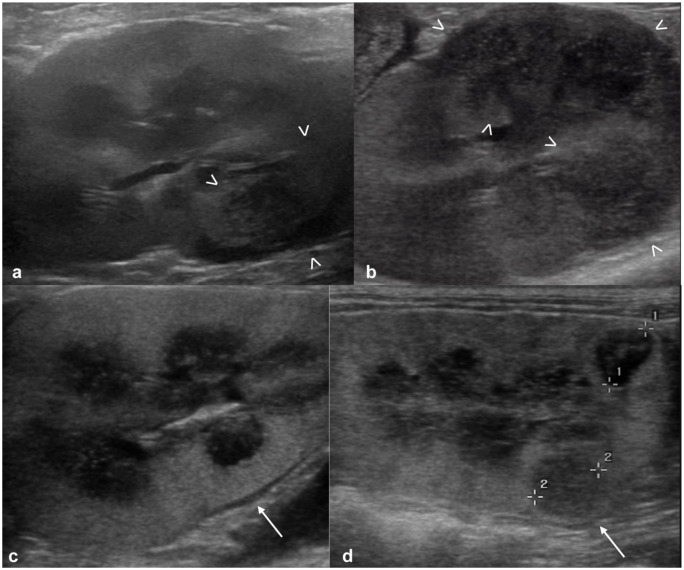
Progressive ultrasonographic findings: (a,c) kidneys of two cats at T0 and (b,d) the same kidneys at T1. In the first cat, an ill-defined hypoechoic mass (between arrowheads) is present at the ventral aspect of the caudal pole at T0 (a) and enlarged at T1 (b). In the second cat, nephromegaly and subcapsular hypoechoic rim (arrow) are visible at T0 (c); in addition, hypoechoic nodules are present at T1, between calipers (d), not present at T0

In two cases, the US findings were worse at T1 compared with T0: in one case, the nodules and/or masses were enlarged compared with previous examination, and in the other, some hypoechoic nodules visible at T1 were not detected at T0 (Figure 6).

Clinical chemistry profiles were available for all cats at T0; 16/24 (67%) cats showed increased creatinine and BUN concentrations above the RIs on presentation. At T1, biochemistry profiles were available for review in 22 cats. Six cats had creatinine and BUN concentrations within the RIs at both T0 and T1 (five with improved US findings and one with static US findings at T1). In 11 cats, the azotemia detected at T0 resolved at T1; four cats were persistently azotemic, with reduction of creatinine and BUN concentrations that remained above the RIs. One non-azotemic cat at T0 became azotemic at T1; this cat also had progressive US findings. In the other cat with progressive US findings, both the renal function markers at T1 were slightly decreased compared with T0 but still above the RIs.

The creatinine and BUN values significantly decreased between T0 and T1 (*P* <0.001 and *P* = 0.004, respectively).

Based on clinical presentation at the oncology appointments, at T1, 21 cats were considered to have experienced positive clinical benefit (20 with improved US findings and one with stable US findings) and two were considered clinically stable (one with stable US findings and one with worsened US findings). One cat was experiencing clinical decline and developed azotemia at T1; this cat had worsened US findings.

The association between US findings, azotemia and clinical presentation is shown in Table 2.

**Table 2:** Association between the ultrasonographic findings, changes in creatinine and blood urea nitrogen concentrations, and clinical findings at T1 (in 22 cats, the clinical chemistry profile was available at T0 and T1)

Ultrasonography	Never azotemic	Azotemia resolved	Azotemia improved but not resolved	Azotemia worsened
Improved	5	10	3	–
	All clinically improved			–
Static	1	1	–	–
	Clinically improved	Clinically static	–	–
Worsened	–	–	1	1
	–	–	Clinically static	Clinically progressive

### US findings at further follow-up

In 17 cats, more than one US follow-up was performed. In eight of these cats, the second follow-up (performed 3–6 months after the diagnosis, still with the ongoing COP protocol) showed worsening of the US findings: most commonly, increased size of the nodules/masses (n = 5), increased size or newly noted hypoechoic rim (n = 2) and reappearance of the resolved nodules and/or masses (n = 1). In four of these cats, clinical chemistry was available at the time of US examination, showing worsened azotemia in three cats and static (within RIs) renal values in one. In the other nine cases, the renal appearance was considered static for more than two follow-ups (6 months to 4 years).

## Discussion

This study described the US characteristics of renal lymphoma after initiating chemotherapy in cats.

Abdominal ultrasonography is routinely used in feline patients with lymphoma for diagnosis, staging and follow-up. Nephromegaly has been described as a common finding in feline renal lymphoma^6,8,9^ and was present in more than 80% of the cats in the current study. In some cases, the presence of multiple nodules or large masses contributed to overall increase of the renal length; a thick subcapsular rim could also significantly affect the size of the kidney, when present. In cases where neither of these two factors was responsible for the increased size and the kidney itself appeared enlarged, a diffuse lymphocytic infiltration was likely the cause of the nephromegaly. Lymphomatous infiltration of kidneys in humans can present as interstitial or intraglomerular; the interstitial form is more common and is frequently associated with nephromegaly and with acute renal failure.^24^ In our study, 67% of cats were azotemic at presentation and all but one had nephromegaly. In humans, one of the theories to explain renal insufficiency in these patients is the increased interstitial pressure, without direct changes in the glomeruli and tubules.^24^ This theory is supported by the rapid recovery in renal function and return to normal size of the kidneys after initiating chemotherapy: renal failure in the case of interstitial lymphoma can occur without tubular apoptosis or necrosis and only secondary to increased pressure and/or ischemia, and can therefore be reversible.^24^ In the intraglomerular form, renal insufficiency is likely caused by an obstruction of glomerular circulation, which is less reversible, and the severity of the disease depends on the proportion of glomeruli affected.^24^ In our population, at the first follow-up after initiating the chemotherapy protocol, only 33% of the cats showed persistent nephromegaly, and most of the patients showed improved or resolved azotemia, possibly suggesting an interstitial form of the disease. Unfortunately, as histology is rarely performed on cats with suspected renal lymphoma, given the possibility of diagnosing lymphoma with a less-invasive fine-needle aspiration, this hypothesis cannot be confirmed. Further studies are necessary to establish if certain US findings, including nephromegaly in the absence of additional findings, are more associated with impaired renal function and with a less favorable response to treatment in cats.

The presence of a subcapsular rim of hypoechoic tissue has been described in cats with lymphoma and was present at diagnosis in 67% of cats included in this study. Valdes-Martinez et al^9^ hypothesized that this subcapsular thickening corresponded to subcapsular lymphomatous infiltrate as opposed to subcapsular effusion, given the evidence of vascularization at color Doppler (also present in our cats, as shown in Figure 3) and similarity to findings described in people. As described in certain human patients with renal lymphoma,^25,26^ our study also demonstrated regression or improvement of the hypoechoic subcapsular rim after chemotherapy, further supporting the hypothesis of lymphomatous infiltration at this level.

Renal lymphoma frequently presented with multiple nodules in our study; this is the most common appearance of renal lymphoma in humans.^27^ Similarly to human lymphoma,^27–29^ these focal lesions were commonly hypoechoic. After treatment, the masses could completely disappear,^30^ as described in some of the cats included in this study. Interestingly, few masses, moderately to markedly hypoechoic at the time of diagnosis, presented as smaller hyperechoic nodules after chemotherapy. The exact reason for this finding is unknown, as no cytology or histology has been performed to assess the underlying pathological changes in the tissue. In humans, hypoattenuating areas are reported on CT in renal lymphomatous masses after treatment,^27^ which may potentially appear hyperechoic on ultrasound. The amount of necrosis, possibly occurring secondary to chemotherapy, the increasing amount of fibrosis and the change in vascular supply could all influence the echogenicity of the mass.

Variable degrees of pelvic distension were detected at the time of diagnosis; this, in conjunction with the retroperitoneal steatitis and effusion, could be considered as a secondary effect of the renal insufficiency rather than a true mechanism of the lymphoma in the kidney. Both signs were less frequently observed after treatment.

The observed improvement at follow-up ultrasonography largely paralleled both the clinical response and the laboratory findings, reflecting the utility of ultrasound as a reliable diagnostic tool to monitor the response to treatment in cases of feline renal lymphoma. The concordance among imaging, clinical signs and biochemical markers underscores the importance of a multimodal approach in the assessment of treatment efficacy. Although CT and positron emission tomography are considered superior for the diagnosis and monitoring of renal lymphoma in human medicine,^27^ the non-invasive nature of ultrasonography makes it especially advantageous for serial monitoring in feline patients.

The large number of patients experiencing a positive clinical response and, on the other hand, the scarcity of worsened cats at first follow-up in this study may not reflect the overall response to chemotherapy and was likely largely influenced by the study design. The focus on US findings led to the exclusion of patients that lacked imaging, possibly because of early death and/or a change in protocol due to clinical worsening.

All included cats underwent a COP-based protocol; however, the retrospective design did not allow standardization in the timing of T1 US examinations. Nevertheless, most of the cats included had T1 US images available at 1 month (and all within 2 months) after starting chemotherapy treatment. A time frame of 4–6 weeks is considered reasonable to expect evidence of US changes and appears convenient with respect to the timing of the COP protocol. At the same appointment, follow-up blood work could be performed to assess the improvement in renal function, which was also often in agreement with US findings. Potentially, US changes may be detectable earlier than 4–6 weeks; unfortunately, none of the included patients had earlier US scans available for review. Further prospective studies are needed to assess better timing for the US follow-up in these patients.

This study has some additional limitations, including the small sample size. A larger number of cases receiving chemotherapy for lymphoma and with US follow-up could have been included if considering lymphoma diagnosed by cytology of other organs with concomitant renal abnormalities; however, the decision of including only confirmed renal lymphoma was made to avoid false positives. From the clinical point of view, when renal lymphoma is part of a multicentric form, US changes of other organs should also be considered to assess the response to treatment, as they could also be responsible for the worsening or persistence of some clinical signs.

## Conclusions

Renal ultrasonography performed after initiating chemotherapy for lymphoma in cats frequently showed a decrease in renal size and reduction or disappearance of the hypoechoic subcapsular rim and of renal nodules. The US findings were frequently in agreement with clinical signs and markers of renal function, highlighting the potential for ultrasonography to provide dynamic information on disease progression or response, thereby informing ongoing therapeutic decisions. In feline renal lymphoma, ultrasonography serves as a valuable tool in both the diagnostic and follow-up phases of patient management.

## References

[1] Mooney SC Hayes AA. Lymphoma in the cat: an approach to diagnosis and management. Semin Vet Med Surg Small Anim 1986; 1: 51–57. 3507786

[2] Mooney SC Hayes AA Matus RE. Renal lymphoma in cats: 28 cases (1977–1984). J Am Vet Med Assoc 1987; 191: 1473–1477. 3693001

[3] Gabor LJ Canfield PJ. Clinical and anatomical features of lymphosarcoma in 118 cats. Aust Vet J 1998; 76: 725–732. 9862061 10.1111/j.1751-0813.1998.tb12300.x

[4] Vail DM Moore AS Ogilvie GK. Feline lymphoma (145 cases): proliferation indices, cluster of differentiation 3 immunoreactivity, and their association with prognosis in 90 cats. J Vet Intern Med 1998; 12: 349–354. 9773411 10.1111/j.1939-1676.1998.tb02134.x

[5] Taylor SS Goodfellow MR Browne WJ. Feline extra-nodal lymphoma: response to chemotherapy and survival in 110 cats. J Small Anim Pract 2009; 50: 584–592. 19891724 10.1111/j.1748-5827.2009.00813.x

[6] Williams AG Hohenhaus AE Lamb KE. Incidence and treatment of feline renal lymphoma: 27 cases. J Feline Med Surg 2021; 23: 936–944. 33464143 10.1177/1098612X20984363 PMC11197130

[7] Walter PA Johnston GR Feeney DA , et al. Applications of ultrasonography in the diagnosis of parenchymal kidney disease in cats: 24 cases (1981–1986). J Am Vet Med Assoc 1988; 192: 92–98. 3277935

[8] Griffin S. Feline abdominal ultrasonography: what’s normal? what’s abnormal? The kidneys and perinephric space. J Feline Med Surg 2020; 22: 409–427. 32326858 10.1177/1098612X20917598 PMC11132528

[9] Valdes‑Martinez A Cianciolo R Mai W. Association between renal hypoechoic subcapsular thickening and lymphosarcoma in cats. Vet Radiol Ultrasound 2007; 48: 357–360. 17691636 10.1111/j.1740-8261.2007.00256.x

[10] Debruyn K Haers K Combes A , et al. Ultrasonography of the feline kidney. J Feline Med Surg 2012; 14: 794–803. 23087005 10.1177/1098612X12464461 PMC11112170

[11] Rossi F Gianni B Marconato L , et al. Comparison of sonographic and CT findings for the identification of renal nodules in dogs and cats. Vet Radiol Ultrasound 2023; 64: 439–447. 36790748 10.1111/vru.13219

[12] Wycislo KL Piech TL. Urinary tract cytology. Vet Clin North Am Small Anim 2019; 49: 247–260. 10.1016/j.cvsm.2018.11.002 30591187

[13] Teske E van Straten G van Noort R , et al. Chemotherapy with cyclophosphamide, vincristine, and prednisolone (COP) in cats with malignant lymphoma: new results with an old protocol. J Vet Intern Med 2002: 16: 179–186. 11899035 10.1892/0891-6640(2002)016<0179:cwcvap>2.3.co;2

[14] Milner RJ Peyton J Cooke K , et al. Response rates and survival times for cats with lymphoma treated with the University of Wisconsin-Madison chemotherapy protocol: 38 cases (1996–2003). J Am Vet Med Assoc 2005; 227: 1118–1122. 16220673 10.2460/javma.2005.227.1118

[15] Versteegh H Zandvliet MJ Feenstra LR , et al. Feline lymphoma: patient characteristics and response outcome of the COP-protocol in cats with malignant lymphoma in the Netherlands. Animals (Basel) 2023; 13. DOI: 10.3390/ani13162667. PMC10451823 37627457

[16] Webster J McNaught KA Morris JS. Evaluation of a multiagent chemotherapy protocol combining vincristine, cyclophosphamide, mitoxantrone and prednisolone (CMOP) for treatment of feline intermediate–large cell lymphoma. J Feline Med Surg 2024; 26. DOI: 10.1177/1098612X241234614. PMC11103311 38647264

[17] Larsen MM Andersson AM Arendt M. Outcome of treatment with a 10-week COP protocol in cats with intermediate or large cell lymphoma: 27 cases (2014–2023). J Small Anim Pract 2024; 65: 807–816. 39113405 10.1111/jsap.13772

[18] Kopecny L Palm CA Skorupski KA , et al. Risk factors associated with progressive increases in serum creatinine concentrations in cats with cancer receiving doxorubicin. J Vet Intern Med 2020; 34: 2048–2055. 32779764 10.1111/jvim.15867 PMC7517847

[19] Peaston AE Maddison JE. Efficacy of doxorubicin as an induction agent for cats with lymphosarcoma. Aust Vet J 1999; 77: 442–444. 10451728 10.1111/j.1751-0813.1999.tb12087.x

[20] Kristal O Lana SE Ogilvie GK , et al. Single agent chemotherapy with doxorubicin for feline lymphoma: a retrospective study of 19 cases (1994–1997). J Vet Intern Med 2001; 15: 125–130. 11300595 10.1892/0891-6640(2001)015<0125:sacwdf>2.3.co;2

[21] Walter PA Feeney DA Johnston GR , et al. Feline renal ultrasonography: quantitative analysis of imaged anatomy. Am J Vet Res 1987; 48: 596–599. 3296881

[22] Nyland TG Widmer WR Mattoon JS. Urinary tract. In: Mattoon JS Nyland TG (eds). Small animal diagnostic ultrasound. 3rd ed. St Louis, MO: Elsevier, 2015, pp 557–607.

[23] Eisenhauer EA Therasse P Bogaerts J , et al. New response evaluation criteria in solid tumours: revised RECIST guideline (version 1.1). Eur J Cancer 2009; 45: 228–247. 19097774 10.1016/j.ejca.2008.10.026

[24] Törnroth T Heiro M Marcussen N , et al. Lymphomas diagnosed by percutaneous kidney biopsy. Am J Kidney Dis 2003; 42: 960–971. 14582040 10.1016/j.ajkd.2003.08.004

[25] Cruz Villalon F Escribano Fernandez J Ramirez Garcia T . The hypoechoic halo: a finding in renal lymphoma. J Clin Ultrasound 1995; 23: 379–381. 7673455 10.1002/jcu.1870230609

[26] Gorg C Weide R Schwerk WB. Unusual perirenal sonographic pattern in malignant lymphoma of the kidney. Clin Radiol 1995; 50: 720–724. 7586967 10.1016/s0009-9260(05)83320-3

[27] Sheth S Ali S Fishman E. Imaging of renal lymphoma: patterns of disease with pathologic correlation. Radiographics 2006; 26: 1151–1168. 16844939 10.1148/rg.264055125

[28] Rohena-Quinquilla IR Lattin GE Jr Wolfman D. Imaging of extranodal genitourinary lymphoma. Radiol Clin North Am 2016; 54: 747–764. 27265606 10.1016/j.rcl.2016.03.009

[29] Charnsangavej C. Lymphoma of the genitourinary tract. Radiol Clin North Am 1990; 28: 865–877. 2190274

[30] Strauss S Libson E Schwartz E , et al. Renal sonography in American Burkitt lymphoma. AJR Am J Roentgenol 1986; 146: 549–552. 3511638 10.2214/ajr.146.3.549

